# The COVID-19 Pandemic and the Migrant Population for HIV Diagnosis and Care Follow-Up: They Are Left Behind

**DOI:** 10.3390/healthcare10091607

**Published:** 2022-08-24

**Authors:** David Zucman, Amina Rasnaama, Catherine Majerholc, Alexandre Vallée

**Affiliations:** 1 Department of Internal Medicine, Reseau Ville Hôpital Val de Seine, Foch Hospital, 92150 Suresnes, France; 2 Department of Epidemiology-Data-Biostatistics, Delegation of Clinical Research and Innovation (DRCI), Foch Hospital, 92150 Suresnes, France

**Keywords:** COVID-19, HIV, migrant people, loss to follow-up, left behind

## Abstract

The coronavirus 2019 (COVID-19) pandemic has posed numerous worldwide challenges. The level of social vulnerability of the migrant population is disproportionately higher than other populations. Recent reports have shown that the access to care for the migrant population (i.e., non-French nationality patients) were greatly impacted during this pandemic. Thus, we would like to highlight the significant impact of the COVID-19 pandemic on care follow-up in those migrant people infected with HIV who receive HIV care in France. Two groups of patients were defined: that is, patients with continuous care and patients with a loss of follow-up of at least one year during the COVID-19 pandemic. Among 672 HIV patients, 19 (2.7%) patients were lost to follow-up for at least one year during the COVID-19 pandemic. We found no significant difference for gender (*p* = 0.332) or age (*p* = 0.115) between the two groups. However, patients with a loss of follow-up were mainly migrants rather than from the other group (*p* < 0.001), and the same results were observed for the nation of birth (89.5% vs. 44%, *p* < 0.001). In our hospital, most of the patients who were living abroad but had HIV care in France before the COVID epidemic (mainly retired migrants) were lost to follow-up during the COVID-19 pandemic. To date, most of them have not resumed HIV care in France and we do not know their present situation. We can only observe that the COVID-19 pandemic has predominately disrupted the HIV care of migrant populations. Do not let them be left behind!

## 1. Introduction

In regard to the COVID-19 pandemic, it is necessary to target people living with HIV to ensure that they continue to have equitable and timely access to healthcare as they are populations that show vulnerability to the consequences of COVID-19 [1]. Previous studies have shown that patients with chronic diseases and conditions, such as HIV status, need additional health support to counteract COVID-19 risk. Moreover, it has been shown that the vulnerability of chronically-ill migrant populations with health issues may be neglected several times [2,3]. This is of concern in migrants (patients with a foreign nationality) living with HIV, especially among those who avoid deportation and who are considered illegal, as immigration laws in several countries do not permit migrants living with HIV to work or stay regardless of their legal status [4].

Migrant populations are considered as vulnerable population groups since they live in overcrowded facilities [5]. Moreover, the number of elderly and disadvantaged migrants remains difficult to estimate. This population is de facto hidden in statistics. In Europe, in 2017, it was estimated that there were between 3.9 and 4.8 million migrants, mainly at risk [6]. Before the COVID-19 pandemic, the migrant population represented around 40% of the new HIV diagnoses in France [7], mainly originating from sub-Saharan Africa [8]. This population consisted mainly of vulnerable undocumented migrants. The coronavirus 2019 (COVID-19) pandemic has posed numerous worldwide challenges [9]. It has disrupted the mobility, socio-economic opportunities, and care of migrant people, especially those who have a precarious status, by exacerbating health vulnerabilities. The level of social vulnerability of this migrant population is disproportionately higher than other populations [6].

France, and Europe, to counteract the COVID-19 pandemic, have been subjected to numerous violations of health rights, the emergence of health inequalities, and the exacerbation of chronic health problems among migrants, especially among those affected by statelessness, who often live in substandard, congested, unhygienic, and unsanitary conditions, or work in informal sectors. There seems to be inequalities for the impact of the COVID-19 pandemic on migrants compared to the general population in Europe [10]. These inequalities did not allow these populations to fully respect public health measures (such as self-isolation/physical distancing/hand sanitation) [11,12]. Due to their living conditions as well as cultural and linguistic obstacles to access information and healthcare, migrant populations are particularly at risk for contracting COVID-19 and other diseases. A recent study reported the significant impact of COVID-19 disease on the first step of human immunodeficiency virus (HIV) care in hospitals in The Netherlands [13]. Using a sensitive tracking network for HIV indicator conditions, they demonstrated a drop in HIV testing rates, test positivity rates, and hospital referrals for new HIV diagnoses. They stated that the temporary closure of public health centers for HIV testing and patients’ delay due to the fear of contracting COVID-19 could explain these findings. Thus, we would like to highlight the significant impact of the COVID-19 pandemic on care follow-up in those migrant people living with HIV who receive HIV care in France.

## 2. Methods

The objective of this study was to investigate the origin profile of HIV patients who were lost to follow-up for at least one year during the COVID-19 pandemic. Loss to follow-up was defined as absence of on-site or remote visit with absence of prescription for antiretroviral therapy made by any means (email, mail, fax). All the people living with HIV followed in our hospital with at least one visit in 2019 were included in the analysis. Age, gender, nationality (French nationality vs. non-French nationality), nation of birth (outside of France vs. France), HIV duration, duration of care in our hospital, and duration of HIV therapy were covered.

Migrant patients were defined as patients with a foreign nationality (i.e., in our study as patients with non-French nationality).

Continuous variables were presented with median (25th–75th percentile) and with number and percentage for categorical variables. Cartography maps were computed with SAS software (version 9.4; SAS Institute, Carry, NC, USA), and comparison between the two groups (i.e., patients with continuous care and patients with loss of follow-up at least one year during the COVID-19 pandemic) were performed with Mann–Whitney test for continuous variables and with Fisher’s test for categorical variables.

The study was approved by the Foch IRB: IRB00012437 (approval number: 22–05-01) on 22 May 2022. Non-opposed consent was obtained for all participants.

## 3. Results

A total of 698 patients living with HIV were included from the Foch hospital, Suresnes, France. Among them, 19 (2.7%) patients showed a loss to follow-up for at least one year during the COVID-19 pandemic. Among the 19 patients with a loss to follow up, all were born in Africa except for two patients born in France.

Among the 19 patients lost to follow-up, 17 patients had their last visit in 2019 and 2 in 2020. After their period of loss to follow-up during the COVID-19 pandemic, only eight patients resumed their follow-up in our hospital in 2021–2022, whereas two patients are known to have been followed in other hospitals in France but without more information, seven patients did not return to care and we do not have any news of them, one patient is known to have died of COVID-19 infection in his country of origin, and one patient went back to France from central Africa, but upon arrival, he presented multiple opportunistic infections and died in France.

**Figure 1:**
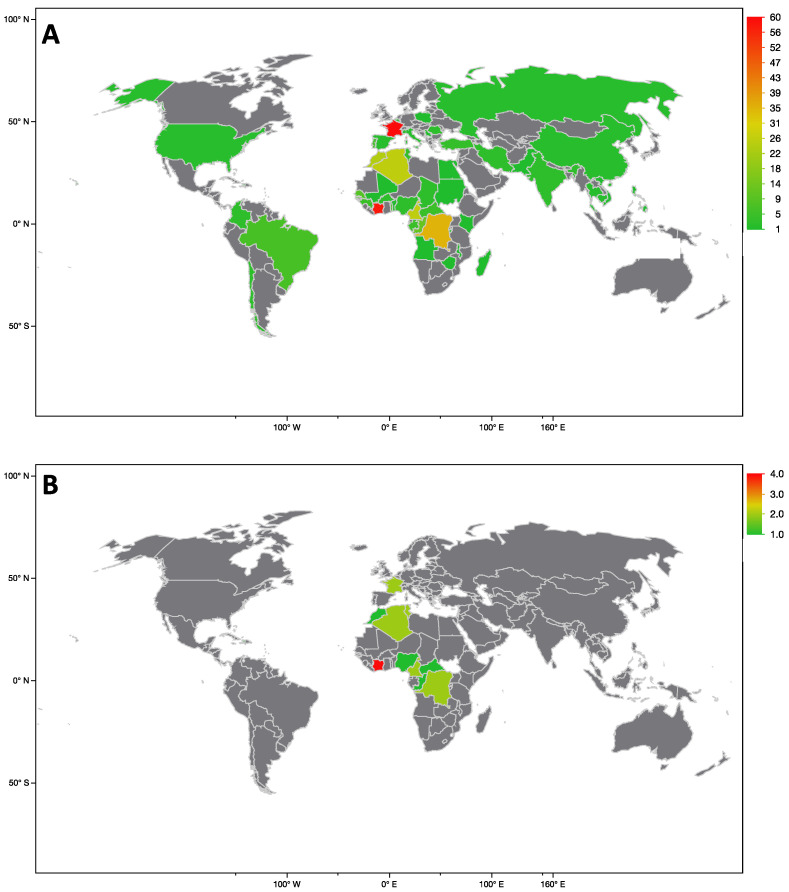
Cartography maps of the recruitment of our hospital of HIV patients according to persistent follow-up (**A**) and loss of follow-up (**B**).

World map 1 shows the repartition of the population with continuous care and World map 2 presents the repartition of the population loss of follow-up during the COVID-19 pandemic (Figure 1a,b).

There was no significant difference between the group of patients with loss of follow-up and the group of patients with continuous care, for gender (*p* = 0.332), age (*p* = 0.115), HIV duration (*p* = 0.736), duration of HIV care in our hospital (*p* = 0.845), and HIV therapy duration (*p* = 0.925). However, patients lost to follow-up were mainly migrants compared to patients with continuous care (79.0% vs. 31.5%, *p* < 0.001). The same results were observed for birth nation (89.5% vs. 44.0%, *p* < 0.001) (Table 1).

In total, 440 patients were male (63.0%), 249 (35.7%) were females, and 9 (1.3%) were transsexuals. Among the 9 transsexuals, all had continuous care during the COVID-19 pandemic.

When comparing only males to females, there was no gender difference for follow-up (*p* = 0.165), but males were older (*p* = 0.009) and there were more with French nationality than women (*p* < 0.001). There was no gender difference with HIV duration (*p* = 0.459), first contact duration with hospital (*p* = 0.430), and therapy duration (*p* = 0.601) (Table 2).

## 4. Discussion

The COVID-19 epidemic has disrupted many aspects of daily life and access to care worldwide. In our short report, we found that few patients stopped their HIV follow-up for more than one year during the COVID-19 pandemic (only 2.7% of the patients). Nevertheless, most of these patients were migrants (loss of follow-up: 79.0% were migrants vs. continuous care: 31.5%, *p* < 0.001) and were not born in France (89.5% vs. 44.0%, *p* < 0.001).

The French hospital database from the regional coordination for HIV (COREVIH) showed a 38.5% drop in new HIV infections reaching hospital care between 2019 and 2020 and a 3.7% drop in HIV-infected patients’ hospital cohorts [14]. Findings, in not yet published work but presented in the French congress on 09/01/2021, from the French National Health Insurance database (called, SNDS), also observed a drop in HIV testing and PrEP initiations during the COVID period. These data showed a 16% decrease in HIV testing between March 2020 and April 2021 and a decrease of around 28% for the same period for PrEP delivery [15].

Retired migrants who returned to their country of origin (mainly North African patients) but maintained an access to care and a temporary housing in France are also a significant part of hospital HIV cohorts in France. In our hospital, most of these retired patients were lost to follow-up for at least one year during the COVID-19 pandemic. Normal access to healthcare has been called into question from the start of the COVID-19 pandemic. Indeed, the French government chose to reduce the spread of the pandemic by introducing different national measures, including lockdown, movement control (through self-attestation), self-isolation, and social distancing [16]. The lockdown and quarantine measures were daunting for migrants living with HIV (with legal or illegal status). The majority of these migrant populations were forced into unemployment while not knowing how to properly access appropriate healthcare, or even access essential medications [1]. In addition, these migrants may have been faced with difficulties in accessing healthcare services on which they previously depended. Patients’ ART prescription duration is 6 months in France. Social security rules limit the length of stay outside of France to periods of 6 consecutive month. It would be convenient to allow longer prescription durations that would permit longer stays in their country of origin, notably for retired migrants, but this would require a modification of the social security rules. Some of them also had to live in inadequate accommodation where they were unable to observe health governmental recommendations, such as safe and appropriate social distancing and hygiene protocols [17]. To date, most of this population has not resumed HIV care in France and we do not know their present situation. In France, the lockdown period between 2020 and 2022 reduced, very significantly, the ability of migrants to access France, either for undocumented or for documented migrants. Flights to and from African countries were closed at different periods for several months. Upon flight re-openings, ticket fares remained very high and prevented some patients from coming back for care. This observation may explain in part the decrease in new HIV diagnoses and in hospital cohorts.

## 5. Conclusions

There is an important need to investigate the life experience of migrants in France to understand the risk of loss to follow-up of the migrant population (non-French nationality) living with HIV, not only for at risk migrants but also for elderly retired migrants, especially during a period of health crisis, such as the COVID-19 pandemic. However, we can only observe that the COVID-19 pandemic has predominately disrupted the HIV care of migrant populations. We must not let them be left behind!

## Figures and Tables

**Table 1:** Characteristics of patients with continuous care compared to patients lost to follow-up (i.e., at least one year during the COVID-19 pandemic).

	Continuous Care N = 679	Loss of Follow-Up N = 19	*p*-Value
Gender (male)	425 (62.6%)	15 (79.0%)	0.332
Non-French nationality (migrants)	214 (31.5%)	15 (79.0%)	<0.001
Birth nation (outside of France)	299 (44.0%)	17 (89.5%)	<0.001
Age (years)	54 (46–62)	58 (51–68)	0.115
HIV duration (years)	19 (10–27)	20 (14–26)	0.736
First contact duration with hospital (years)	14 (6–21)	11 (7–21)	0.845
Therapy duration (years)	16 (9–23)	18 (9–25)	0.925

**Table 2:** Characteristics of females and males of the study population.

	Females N = 249	Males N = 440	*p*-Value
Lost to follow-up	4 (1.61%)	15 (3.41%)	0.165
Non-French nationality (migrants)	126 (50.6%)	101 (23.0%)	<0.001
Birth nation (outside of France)	175 (70.3%)	138 (31.4%)	<0.001
Age (years)	52 (45–59)	56 (47–63)	0.009
HIV duration (years)	19 (12–26)	18 (10–27)	0.459
First contact duration with hospital (years)	15 (7–21)	14 (9–24)	0.430
Therapy duration (years)	16 (10–23)	15 (9–24)	0.601

## Data Availability

The data that support the findings of this study are available on request from the corresponding author. The data are not publicly available due to privacy or ethical restrictions.
